# Oral health-related multiple outcomes of holistic health in elderly individuals: An umbrella review of systematic reviews and meta-analyses

**DOI:** 10.3389/fpubh.2022.1021104

**Published:** 2022-10-27

**Authors:** Fan Liu, Siping Song, Xin Ye, Shuqi Huang, Jing He, Guan Wang, Xiuying Hu

**Affiliations:** ^1^West China School of Nursing, State Key Laboratory of Oral Diseases & National Clinical Research Center for Oral Diseases, West China Hospital of Stomatology, Sichuan University, Chengdu, China; ^2^Innovation Center of Nursing Research, Nursing Key Laboratory of Sichuan Province, West China Hospital, Sichuan University, Chengdu, China

**Keywords:** oral health, holistic health, elderly individuals, multiple outcomes, umbrella review

## Abstract

**Background and aims:**

Along with an aging population, exploring the impact of oral health on holistic health and determining exact outcomes in elderly individuals are important in both scientific research and clinical practice. Significant increase in the number of systematic reviews shows that oral health can directly or indirectly affect the overall health of elderly people physically, mentally and socially. To systematically collate, appraise, and synthesize the current evidence, we carried out an umbrella review of the impacts of oral health on holistic health in elderly individuals.

**Methods:**

A systematic reviews and meta-analyses search was performed in the major databases PubMed, MEDLINE, Web of Science and the Cochrane Library from inception to February 1, 2022, according to the Preferred Reporting Items for Systematic Reviews and Meta-Analyses (PRISMA) statement. The JBI (Joanna Briggs Institute) Critical Appraisal Checklist for Systematic Reviews and Research Syntheses was referred to assess methodological quality, and the GRADE (Grading of Recommendations, assessment, Development, and Evaluation working group classification) was used to assess the quality of evidence for each outcome included in the umbrella review.

**Results:**

Out of 1,067 records, a total of 35 systematic reviews were included. Respiratory diseases, malnutrition, age-related oral changes, frailty, cognitive impairment, depression and poor quality of life were identified as seven key outcomes that affect the physical, mental and social health of elderly individuals. Meanwhile, three intervention measures of oral health were summarized as (i) more rigorous and universal scales, (ii) dental cleaning and denture installation, and (iii) improving self-awareness regarding oral care.

**Conclusions:**

Evidence showed that oral health can significantly affect holistic health, and the diverse oral diseases directly lead to multiple health outcomes in elderly individuals. Clear high-quality evidence revealed that oral health is strongly associated with seven health outcomes covering physical, mental, and social levels, which directly corresponds to holistic health, and impacts the quality of life of elderly individuals. Such the results remind the importance of oral care in public health, and further studies need to be conducted to verity more specific association between oral health and other chronic diseases.

**Systematic review registration:**

https://www.crd.york.ac.uk/PROSPERO/#recordDetails, identifier: CRD42022315315.

## Introduction

With the rapid aging of the population worldwide, the holistic health of elderly individuals has become a crucial issue in both developing and developed countries (1, 2). In general, holistic health is defined as a state of complete physical, mental and social functioning, not only the absence of disease and frailty (3), which has been increasingly studied, especially among older people. With increased life expectancy (4), the risks of multiple chronic communicable/non-communicable physical diseases, mental impairments and social disorders are increasing continuously (5, 6). In addition, people have become increasingly aware that the occurrence of such diseases tends to be a cross result; namely, there is always a strong correlation between one poor health outcome and multiple complications, which, however, is severely neglected (7). Typically, as one of the most crucial issues among elderly individuals, people realize that poor oral health is significantly associated with holistic health and can result in multiple health outcomes (8–11). Furthermore, instead of primarily addressing oral diseases or oral hygiene itself, an increasing number of studies in recent years have focused on revealing the correlation between oral health and different common diseases in elderly individuals (12–14).

For instance, evidence has shown that 15% of cases of community-acquired pneumonia or other respiratory diseases, which are considered some of the most common chronic diseases among elderly individuals, are significantly associated with oral bacteria (15). Correspondingly, it has been reported that once regular oral nursing care is lacking, the pathogenic microorganisms colonizing the oral microbiome are strongly correlated with the aggravation of respiratory diseases, including aspiration pneumonia, chronic obstructive pulmonary disease (COPD), and even COVID-19, in elderly individuals in residential care (16–18). Simultaneously, older people with poor awareness of autonomous oral care not only take higher risks of periodontitis and/or dental caries related to oral diseases, but the higher load of microorganisms easily gives rise to frailty (11, 16), loss of function (19) and multiple chronic comorbidities (20, 21). Tooth loss and a decline in chewing ability are also considered to be accompanied by malnutrition and low quality of life (22, 23). In addition, poor oral health can directly and indirectly impact the mental health of elderly individuals and lead to cognitive impairment (24) and depression (25, 26). Moreover, it has also been reported that understanding the economic burden of oral diseases is essential to evaluate the societal relevance of preventing and addressing oral diseases (27).

Although there is an increasing amount of research and systematic reviews on the impacts of oral health on different health outcomes, and published articles on corresponding topics have covered a variety of general health outcomes among elderly individuals, such studies have mainly focused on the association between oral health and some specific diseases; a body of work describing the current landscape of intervention and nursing measures that links oral health and the diverse outcomes covering physical, mental, and social health is still missing (28, 29). Accordingly, it is still challenging for practitioners and decision-makers to find, interpret, and utilize related evidence in daily nursing and medical processes.

The aim of this umbrella review, therefore, is to provide an overall examination and synthesis of the available qualitative and quantitative evidence on the oral health of elderly individuals from a higher-level perspective and explain the impact of oral health on multiple outcomes of holistic health among elderly individuals. On this basis, the objectives of the review are to assess, identify, analyse, and synthesize the evidence on (1) an exact correlation between oral health and multiple outcomes of holistic health in older people and (2) solutions and suggestions, including effective interventions in clinical practice for nursing care.

## Materials and methods

The protocol of this study was registered in PROSPERO registry (CRD42022315315), and the review is reported in accordance with the Preferred Reporting Items for Systematic Reviews and Meta-Analyses guidelines (PRISMA).

### Eligibility criteria

Studies were selected based on the following eligibility criteria: (a) included a systematic review with/without meta-analyses on oral health among older people; (b) investigated the association of different multiple outcomes of holistic health among older people; and (c) included older people as the study population, which exactly corresponds to populations, interventions, comparators, outcomes and study designs (PICOS) as presented in Table 1.

**Table 1:** PICOS criteria for inclusion of studies.

**Item**	**Criteria**	**Description**
Participation	Does the study involve elderly individuals?	Yes
Intervention	NA	
Comparator	NA	
Outcomes	Does the study report effect on oral health-related multiple outcomes of holistic health?	a. Respiratory diseases b. Malnutrition c. Frailty d. Diabetes mellitus e. Cardiovascular conditions f. Age-related oral changes g. Cognitive impairment h. Depression i. Poor quality of life
Study design	Is the study a systematic review?	Systematic reviews with/without meta-analyses are considered.

NA, not available.

### Data sources and search strategy

The electronic databases PubMed, MEDLINE, Web of Science, and the Cochrane Library were systematically searched for review articles to identify systematic reviews with meta-analyses of observational or interventional studies that researched the multiple oral health-related outcomes of holistic health among older people. The following research strategy was used to conduct the literature search: (oral health or oral diseases) AND (systematic review* OR meta-analysis^*^), using truncated terminology for all areas (see Supplementary Table 1). We limited search to reviews written in English or Chinese published before February 2022. Searches were restricted by the level of evidence (systematic review and meta-analysis, or other evidence syntheses). Literature reviews or other types of non-systematic reviews were excluded. In addition, we checked reference lists and performed lateral searches using the “related articles” option in PubMed and the “cite by” option in Web of Science. The reference lists of eligible papers and relevant clinical guidelines were also searched (see research strategy in Supplementary Table 1). All searches were carried out on 1 February 2022.

### Selection of reviews

Two of the authors (FL and SS) worked together to remove duplicates with EndNote reference management software, reviewed titles and abstracts for relevance, and finally selected the papers, which required the agreement of both reviewers. Disagreements were resolved through consensus or discussion with the third researcher (XH).

### Quality appraisal

Review articles included in the final analysis were critically evaluated using the “JBI Critical Appraisal Checklist for Systematic Reviews and Research Syntheses” (JBI CAC for SRRS) by two reviewers (30), and no reviews were eliminated according to their results from this instrument. The JBI assessment tool consists of 11 questions (see the detailed checklist in the Supplementary material). Every question is assessed with four possible answers: Yes, No, Unclear or Not Applicable. Each “Yes” response gains one point, and all other answers receive zero points. Based on the sum of the points, the quality of papers was divided into three groups: low (0–4), moderate (5–8), and high (9–11) quality (31). The quality of the evidence (QoE) supporting each bottom-line statement was rated by using a method of the GRADE approach for primary evidence (1 = very low; 2 = low; 3 = moderate; 4 = high) (Box 1) (32, 33). This method takes into account the study design (meta-analysis; yes or no) and JBI CAC rating of the included systematic reviews.

Box 1 **Method used to rate the quality of the evidence supporting each bottom-line statement.**

**Figure:**
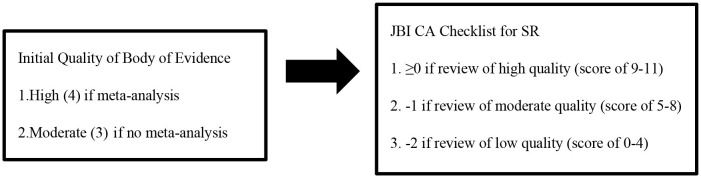

CA, critical appraisal; SR, systematic reviews.

### Data extraction and synthesis

The following data were extracted from each manuscript: (1) the name of the first author and publication year; (2) the study design, number of primary studies included in the meta-analysis, the study characteristics and the total number of participants; (3) the respective outcome according to the eligibility criteria; and (4) risk estimates and corresponding confidence intervals and the respective significance level. If relevant data were not available in the respective papers, we sent email inquiries to the corresponding authors. If the author did not reply or could not provide the missing data, we marked the missing information as “No”. Reviews and primary studies were classified as quantitative, qualitative or mixed methods studies. The primary studies in each review were tabulated to assess the overlap between reviews. Effectiveness was investigated by categorizing the different outcomes reported and tabulating the data, including an indication of whether the effects of the intervention were positive, negative or not statistically significant.

## Results

### Selection of relevant reviews

**Figure 1:**
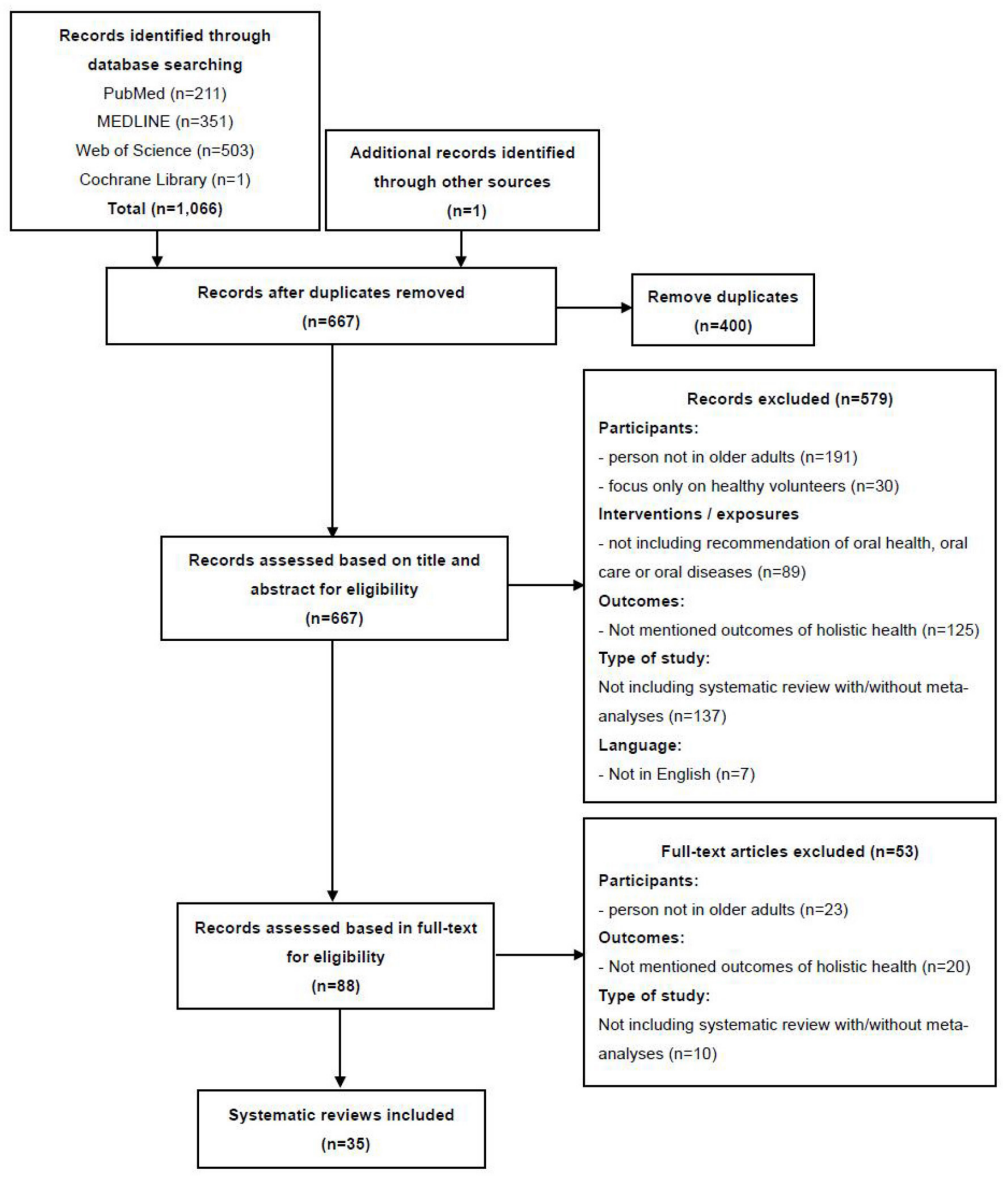
PRISMA flowchart.

A total of 1,066 records identified through database searching and 1 additional record identified through other sources were imported into the reference management programme EndNote X9.3.1. After examining the records in this programme, 400 duplicates were removed, and 667 records remained. Next, two authors independently screened the titles and abstracts of the remaining records, and 579 papers were excluded because they did not meet the inclusion criteria. Furthermore, based on the inclusion and exclusion criteria, 53 of the 88 articles were excluded after full-text review because these studies did not focus on elderly individuals, did not mention outcomes of holistic health, or did not include systematic reviews with/without meta-analyses. Finally, 35 review papers met the inclusion criteria and were included in the analysis and synthesis of this study. Figure 1 illustrates the detailed selection of the review papers. The detailed information of the papers is shown in Table 2, which summarizes the elements of the selected reviews, indicating the diversity of research designs, participant groups and outcomes addressed by the authors.

**Table 2:** Main characteristics of the articles included in the systematic umbrella review.

**References, review design**	**Aim or objective**	**Search strategy**	**Type of studies: total number + design**	**Setting of participants. Total number of participants (if available)**	**Study outcomes related to holistic health**
**Nutrition**					
Algra et al. (34) Systematic review	To examine the association between malnutrition and oral health in older people.	Databases: 4, PubMed, CINAHL, DOSS, and Embase. Langue: limited to English and Dutch. Date: from January 2000 to May 2020.	10 studies: 9 cross-sectional studies + 1 longitudinal cohort study	Nursing homes, dental clinics, community community-welling older people, or hospital units (acute care units or rehabilitation). Total *N* = 9,093.	The association between malnutrition and oral health, the state of hard and soft tissues of the mouth, salivary flow, and xerostomia
Hussein et al. (28) Systematic review and meta-analysis	To evaluate whether poor oral health is associated with a higher risk of malnutrition based on MNA or MNA-SF in older adults.	Databases:4, PubMed, Web of Science, Cochrane Library and EMBASE. language: English. Date: up to June 2020.	33 studies: 28 cross-sectional studies + 5 cohort studies	Community-dwelling older adults, interviewed at home or in emergency rooms or hospital wards, institutionalized older adults, living in nursing homes or assisted living facilities. Total *N* = 27,559	MNA or MNA-SF
Tada and Miura (35) Systematic review	To assess the association of mastication with food and nutrient intake in the community dwelling elderly.	Databases:4, PubMed, Web of Science, Cochrane Library, and Scirus. Langue: English. Date: between 1991 and 2013.	35 studies: 28 cross-sectional studies + 7 intervention studies	Community dwelling elderly (Non-institutionalized healthy elderly, dental school hospitals.) Total *N* not reported.	Food intake or nutrient intake
Toniazzo et al. (36) Systematic review and meta-analysis	To compare the nutritional status and oral health in older individuals.	Databases:3, Medline-Pubmed, Scopus and EMBASE. Language: no restrictions. Data: up to October 28th 2016.	26 studies: 23 cross-sectionals + 1 case-control + 1 cohort + 1 clinical trial	Non-institutionalized older adults. Total *N* = 13,257.	The malnutrition or risk of malnutrition related with oral health
Van Lancker et al. (37) Systematic review	To determine whether an association exists between oral health and malnutrition in the elderly in a long-term care facility.	Databases:3, Medline, CINAHL and Cochrane Library. Language: in English, Dutch, French, or German. Date: between January 1985 and May 2011.	16 cross-sectional studies	Long-term care facility. Total *N* not reported, number of included patients varied between 40 and 3,088.	The association between oral health status and malnutrition
Zelig et al. (38) Systematic review and meta-analysis	To determine the association between tooth loss and nutritional status by the validated nutrition screening or assessment tool.	Databases:5, PubMed, Scopus, CINAHL, Web of Science, and MEDLINE. Language: English. Date: between January 2009 and December 2019.	7 studies: 6 cross-sectionals and 1 cohort	Adults living in the community and institutions. Total *N* = 7,897.	Associations between tooth loss and nutritional status
**Age-related oral changes**					
Affoo et al. (39) Systematic review and meta-analysis	To determine whether salivary flow decreases as a function of aging.	Database:7, PubMed, EBSCOhost, Web of Science, Cochrane library, EMBASE, Dissertations and Theses, and Scopus databases. Language: English. Date: up to June 2013.	47 cohort studies	All general elderly. Total *N* not reported, number of included patients varied between 15 and 1,427.	The aging process is associated with reduced salivary flow in a salivary-gland.
Pina et al. (40) Systematic review and meta-analysis	To determine the prevalence of hyposalivation in older people.	Database:6, Embase, LILACS, Medline, PubMed, Web of Science and Google Scholar. Language: no restrictions. Date: up to February 2019.	13studies: 10 cross-sectional studies + 3 cohorts	All general elderly. Total *N* = 3,885	The prevalence of hyposalivation
Ruiz-roca et al. (41) Systematic review	To ascertain the oral health status of older people patients admitted to institutions or hospitalized for a long period of time.	Database: 2, PubMed and Cochrane Library. Language: English and/or Spanish and/or Portuguese. Date: 1 January 2014 to 1 January 2019.	5 studies: 2 cross-sectional + 2 cohorts + 1 case-control study	Older hospitalized for long term at least 7 days. Total *N* = 773	Poorer results in patients institutionalized in the periodontal index score
Chan et al. (42) Systematic review	To provide an update on caries prevalence in older adults around the globe.	Database: 5, PubMed, Scopus, Embase/Ovid and Web of Science, Google Scholar. Language: English. Date: January 2016 to March 2021.	39 (8 additional) cross-sectional surveys	Community, residential homes, and hospitals. Total *N* not reported.	Caries prevalence
López et al. (43) Systematic review	To review the burden of caries and periodontitis in the elderly, changes with age that can explain this burden, and the vulnerability to disease of elderly populations.	Database: 2, PubMed, Medline. Language: NA.	18 studies	All general elderly. Total *N* not reported.	Caries and periodontitis burden
**Quality of life**					
van de Rijt et al. (44) Systematic review	To identify oral health factors associated with OHQoL in the elderly and to give a comprehensive overview of the body of literature for each oral health factor separately.	Databases:5, PubMed, Embase, EBSCO/CINAHL, EBSCO/PsycINFO, and Wiley/ Cochrane Library. Language: no restrictions. Date: up to April 3, 2019.	68 studies: 9 RCTs (randomized clinical trials) + 6 cohort studies + 53 cross-sectional studies	In community or in institutional care. Total *N* = 47,770.	OHQoL
Wong et al. (45) Systematic review	To describe recently reported oral health levels, the OHRQoL and the associated factors.	Databases:6, Cochrane Library, JBI, MEDLINE, EMBASE, CINAHL, Google Scholar. Language: English. Date: between July 2009 and June 2019.	25 studies: 22 cross-sectional studies + 3 case-control studies	Institutionalized homes, long-term care, nursing homes and residential homes. Total *N* = 10,958.	OHQoL
Azami-Aghdash et al. (46) Systematic review and meta-analysis	To assess oral health- related quality of life in older people.	Database: 7, PubMed, EMBASE, Scopus, SID, MagIran, Cochrane Library, google scholar. Language: English. Date: from 1st Jan 2000 to 30th Jan 2017.	40 studies: The type of included studies was not mentioned	All general elderly. Total *N* = 22,416.	OHQoL
Baniasadi et al. (47) Systematic review and meta-analysis	To determine the relationship between poor OHRQoL and oral health determinants.	Database: 4, PubMed, Scopus, Cochrane Library and Web of Science. Language: English. Date: before 1 December 2019.	19 studies: 18 cross-sectional studies + 1 cohort study	All general elderly. Total *N* not reported.	The association between poor OHRQoL and oral health.
de Medeiros et al. (48) Systematic review	To evaluate whether treatment with new complete dentures improves quality of life in elderly patients.	Database: 6, MEDLINE/PubMed, Scopus, LILACS, SciELO, Web of Science, and Cochrane Library. Language: in English, Spanish, Portuguese. Data: up to March 2018.	7 studies: 6 RCTs + 1 cohort study	Residential or nursing homes, sheltered accommodations, private homes, or hospitals. Total *N* = 618.	Quality of life in elderly patients with new complete dentures
Ming et al. (49) Systematic review and meta-analysis	To increase understanding of OHRQoL among patients with AD and explore factors that may affect OHRQoL.	Database: 6, PubMed, Cochrane Library, Medline, EBSCO, ProQuest, and EMBASE. Language: English. Date: until August 30, 2018.	6 studies: 5 cross-sectional designs + 1 nRCT	All general elderly. Total *N* = 632.	The association between OHRQoL and AD
**Respiratory diseases**					
Azarpazhooh et al. (50) Systematic review	To investigate evidence for a possible etiological association between oral health and pneumonia or other respiratory diseases.	Database: 9, Ovid MEDLINE, Cumulative Index to Nursing & Allied Health Literature, Cochrane library, Database of Abstracts of Reviews of Effects, EMBASE, Health and Psychosocial Instruments, HealthSTAR, PubMed, and Google Scholar. Language: English. Date: from the earliest record until July 2005.	10 studies: 9 case-control + 1 cohort study	In nursing homes and especially in ICUs. Total *N* not reported.	Outcomes related to respiratory diseases
Khadka et al. (13) Systematic review	To determine potentially pathogenic microorganisms in oral specimens of older people with aspiration pneumonia and the effect of POHC in reducing aspiration pneumonia risk.	Database:7, PunMed/MEDLINE, CINAHL, EMBASE, Cochrane library, PROQUEST, Google Scholar, and Web of Science. Language: English. Date: published between January 2001 and December 2019.	12 studies: 4 cross-sectionals + 5 cohort + 3 interventions (9 observational studies included a followed-up period ranging from 6 months to 9 years or until participants were no longer living.	Older adults in residential care. Total *N* not reported.	Aspiration pneumonia as an outcome and describing oral microorganisms.
Liu et al. (51) Systematic review and meta-analysis	To assess effects of oral care measures for preventing nursing home-acquired pneumonia in residents of nursing homes and other long-term care facilities.	Database: 6, Cochrane Library, MEDLINE Ovid, Embase Ovid, CINAHL, ClinicalTrails. Gov, WHO. Language: no restrictions. Date: up to 15 November 2017.	4 RCTs	Residents of nursing homes and other long-term care facilities. Total *N* = 3,905.	Outcomes related to home-acquired pneumonia.
Loeb et al. (52) Systematic review	To assess the effectiveness of prevention of AP in older adults: compensatory strategy/positioning changes, dietary interventions, pharmacologic therapies, oral hygiene, and tube feeding.	Database:5, MEDLINE, EMBASE, Cochrane Library, CINAHL, and HealthSTAR databases. Language: no restrictions. Date: from 1997 to 2001.	8 RCTs	Older adults in nursing homes. Total *N* = 1,234.	Primary outcome: AP; Secondary outcomes: related to the effects of aspiration that were assessed included dehydration, gross aspiration, nutritional intake, and death.
Scannapieco et al. (15) Systematic review	To determine if interventions that improve oral hygiene reduce the rate of pneumonia in high-risk population.	Database: 4, MEDLINE, pre-MEDLINE, MEDLINE Daily Update, and Cochrane library. Language: English. Date: from 1966 through March 2002.	30 studies: 21 case-control studies + cohort studies + 9 RCTs.	Elder patients or institutionalized patients in nursing homes. Total *N* = 3,172.	Primary outcome: reduced rate of pneumonia or lung disease, Secondary outcomes: improved lung function. Patients-centered outcomes: quality life issues, Adverse outcomes: intraoral adverse effects, increased rate of pneumonia.
Sjögren et al. (53) Systematic review and meta-analysis	To compare the effect of intensified oral care interventions given by dental or nursing personnel on mortality in elderly adults in hospitals or nursing homes with the effect of usual oral care.	Database: 3, PubMed, Cochrane library, and HTA. Language: English, Danish, Norwegian, or Swedish. Date: published since January 1, 1996.	5 RCTs	Elderly adults in hospitals or nursing homes. Total *N* not reported.	The outcome was mortality from HAP
Sjögren et al. (54) Systematic review	To investigate the preventive effect of oral hygiene on pneumonia and respiratory tract infection, focusing on elderly people in hospitals and nursing homes.	Database: 2, Medline, Cochrane library. Language: Dutch, English, German, and any of the Nordic languages (Danish, Finnish, Icelandic, Norwegian, Swedish). Date: from 1996 to 2007.	15 studies: 5 RCTs + 10 nRCTs	Elderly people in hospitals and nursing homes. Total *N* not reported.	Pneumonia and respiratory tract infection, death from pneumonia
van der Maarel-Wierink et al. (55) Systematic review	To systematically review the risks for aspiration pneumonia in frail older people and the contribution of bad oral health among the risk factors.	Database: 5, PubMed (Medline), Web of Science, Cochrane Library, EMBASE, and CINAHL. Language: English. Date: January 2000 to April 2009.	21 studies: 1 case-cohort + 12 cohort + 7 case-control + 1 cross-sectional design	Frail elder people in hospitalized, institutionalized, or frail home-dwelling. Total *N* not reported.	The risks of aspiration pneumonia
van der Maarel-Wierink et al. (56) Systematic review	To systematically review the literature on oral health care interventions in frail older people and the effect on the incidence of aspiration pneumonia.	Database: 5, PubMed, Web of Science, Cochrane Library, EMBASE and CINAHL. Language: English. Date: from January 2000 to July 2010.	5 RCTs	Hospitalized or institutionalized people. Total *N* not reported.	The incidence of aspiration pneumonia
**Frailty**					
Slashcheva et al. (29) Systematic review	To updating the evidence for association between oral health characteristics and frailty status, identifying gaps in translational dental research and application of frailty assessment into clinical practice.	Database: 5, MEDLINE and Epub Ahead of Print, In- Process & Other Non-Indexed Citations and Daily, Embase, Cochrane library and Scopus. Language: English. Date: Until to 2 January 2020.	26 studies: 17 Cross sectional studies + 9 longitudinal studies	All general elderly. Total *N* = 37,925.	Association between oral health characteristics and frailty status
Tôrres et al. (57) Systematic review	To assess the relationship between frailty or one of its components and poor oral health.	Database: 5, PubMed, EMBASE, Cochrane library, LILACS, and SciELO. Language: English, Spanish, or Brazilian Portuguese. Date: from 1991 to July 2013.	12 studies: 7 cross-sectional + 5 cohort studies.	The frail elderly. Total *N* = 7,884.	Different oral health predictors and covariates that are associated with frailty or its components.
Hakeem et al. (58) Systematic review	To systematically review longitudinal studies on the association between oral health and frailty indicated by any validated scale or index.	Database: 3, MEDLINE, EMBASE and LILACS. Language: no restriction. Date: up to July 2018.	5 longitudinal studies	No considerations were given for the recruitment settings (nursing or care homes, hospitals, community dwelling), nor the general health status. Total *N* not reported.	Association between oral health and frailty
**Cognitive impairment**					
Nangle et al. (59) Systematic review	To focus on how oral health relates to specific cognitive abilities in older adults.	Database: 4, PubMed, Scopus, PsychINFO, and Web of Science. Language: English. Date Until August, 2018.	23 studies: 19 cross sectionals + 3 longitudinal + 1 interventional	NA Total *N* = 121,547.	Relationship between oral health and cognitive decline in late adulthood
Lauritano et al. (60) Systematic review	To present a literature review of oral health status and the need for oral care in people with dementia, as compared to people without dementia and also of the relationship between periodontal disease and cognitive impairment.	Database: 3, PubMed, CINAHL, and Cochrane Library. Language: no restrictions. Date: within 2019.	56 studies: 19 cohort + 9 case-control + 26 cross-sectional + 2 RCTs	Participants must have been diagnosed with dementia, and should be 60 years or older. Total *N* = 15,263.	Role of tooth loss due to periodontal disease in progression of dementia.
Delwel et al. (61) Systematic review	To provide a comprehensive literature overview following a systematic approach of the level of oral hygiene and oral health status in older people with dementia with focus on oral soft tissues.	Databases: 3, PubMed, CINAHL, and the Cochrane Library. Language: Dutch, English, and German. Date: performed on 12 January 2017.	36 studies: 14 cross-sectional studies + 10 case-control studies + 10 + cohort studies + 2 RCTs	Nursing homes, Community, Non-institutionalized, institutionalized, assisted living, acute bed geriatric hospital, psychiatric hospital, long-term care homes. Total *N* = 9,182.	Oral hygiene and oral health status
Delwel et al. (62) Systematic review	To provide a systematic overview including a quality assessment of studies about oral health and orofacial pain in older people with dementia, compared to older people without dementia.	Database: 3, PubMed, CINAHL, and the Cochrane Library. Language: no restrictions. Date: up to now.	37 studies: 11 cohort + 6 case-control studies + 19 cross-sectional + 1 RCT	All general elderly. Total *N* = 7,806.	Oral health status
Wu et al. (63) Systematic review	To systematically review longitudinal studies examining the association between oral health and cognitive decline.	Database:3, PubMed, Medline, CINAHL. Language: English. Date: January 1993 and March 2013.	56 studies: 40 cross-sectional studies + 16 longitudinal studies	All general elderly. Total *N* not reported.	The risk of cognitive decline or incident dementia
**Depression**					
Cademartori et al. (64) Systematic review and meta-analysis	To systematically review the literature in order to investigate association between depression and oral diseases.	Database: 5, PsychInfo, PubMed, Scielo, Scopus, and Web of Science. Language: no restriction Date: until 20 April 2018	15 studies: 14 cross-sectional studies + 1 longitudinal study	All general elderly. Total *N* not reported.	Dental caries/periodontal disease/tooth loss

AD, Alzheimer's disease; AP, aspiration pneumonia; DOSS, Dentistry and Oral Sciences Source; MNA, mini nutritional assessment; MNA-SF, mini nutritional assessment short form; NA, not available; OHRQoL, oral health-related quality of life; POHC, professional oral hygiene care.

### Participants and settings

Six of the included papers were from the Netherlands, five were from the United States, five were from Brazil, four were from China, three were from Canada, two were from Australia, Iran and Sweden, and one was from the United Kingdom, Italy, Japan, Spain, Denmark and Belgium, respectively. The average number of authors was 5.3 ± 1.9. The publishing year of the papers ranged from 2003 to 2021. The average number of databases referred was 4.5 ± 1.6.

The most common groups of participants investigated were elderly individuals living in hospitals or nursing homes. Meanwhile, community-dwelling or institutionalized/non-institutionalized older adults were also the main participant groups investigated in the primary studies. In addition, three studies considered all elderly individuals in general. Among the included reviews, total participants ranged from 618 to 121,547, while 13 of the included papers did not report the total number but presented the ranges of the ages of the elderly participants instead.

### Designs and characteristics of the reviewed articles

Most of the included systematic reviews consisted of cross-sectional and longitudinal studies, and 12 reviews consisted of randomized controlled trials (RCTs). The summarized findings of the included reviews came from between 4 and 68 primary studies. Eleven reviews also conducted meta-analyses with/without RCT results that were relevant to the present umbrella review.

### Critical assessment and risk of bias in the included review papers

The corresponding detailed critical assessment and risk of bias of the reviews are shown in Supplementary Table 2. Eight papers were evaluated as medium quality, and the others were graded as high quality.

### Key data synthesis results

The included papers showed a varied range of the impacts of oral health on holistic health among older people. According to the definition of holistic health, three secondary level subthemes can be conceptualized to meaningfully generalize the comprehensive outcomes.

In terms of the research topics, the subthemes based on the main theme can be identified as (i) physical outcomes (*N* = 23); (ii) mental outcomes (*N* = 6); and (iii) social outcomes (*N* = 6), as shown in Supplementary Table 3.

### Physical outcomes

Twenty-three systematic reviews provided data on physical outcomes, of which 7 included a meta-analysis. Nine systematic reviews (2 with a meta-analysis) (29, 30) focused on respiratory diseases (13, 15, 50–56), 5 (2 with a meta-analysis) (39, 40) on age-related oral changes (39–43), 6 (3 with a meta-analysis) (28, 36, 38) mainly on malnutrition and 3 (none with a meta-analysis) on frailty (29, 57, 58).

#### Respiratory diseases

All the corresponding reviews, in general, showed that oral health is crucially related to the incidence of aspiration pneumonia, and various poor oral hygiene factors are considered significant risk factors for different respiratory diseases. Five systematic reviews with low to high quality scores showed that the prevalence of cariogenic and periodontal pathogens in saliva and dental plaque as well as dental decay due to poor oral hygiene are potential risk factors for pneumonia and chronic obstructive pulmonary disease (13, 15, 50, 55, 56). One systematic review of moderate quality pointed out that higher plaque scores were strongly associated with a previous history of respiratory tract infection (50). In particular, one moderate-quality review mentioned that the detection and isolation rates of *Candida albicans, Staphylococcus aureus*, methicillin-resistant *S. aureus, Pseudomonas aeruginosa*, and even *Escherichia*, which are strongly related to mortality due to aspiration pneumonia, are much higher among older people with poor oral health (13).

On the other hand, 6 systematic reviews (2 with meta-analyses) of moderate to high quality showed that regular oral care intervention and cleaning are significant factors for preventing aspiration pneumonia in older people (13, 15, 51–54). Mechanical and/or topical chemical disinfection or antibiotics such as povidone iodine can greatly reduce the incidence of nosocomial pneumonia (15, 52). Both the meta-analyses of high quality examined the impacts of oral hygiene on the incidence rate of hospital aspiration pneumonia (HAP) and showed that while there was no evidence verifying whether professional oral care was better or worse than routine oral care, it demonstrated that professional oral care can truly reduce mortality from HAP (51, 53). One review of low quality pointed out that tooth brushing, denture cleaning and regular professional oral health care are the best interventions to reduce the incidence of aspiration pneumonia (56). Combined with the quality and results of the included reviews, respiratory diseases should be considered to be closely associated with poor oral health.

#### Age-related oral changes

Poor oral health adds to the burden of the change in the microenvironment for older people (43), which directly aggravates dental/root caries and periodontitis in both developing and developed countries among institutionalized elderly individuals (42). Two high-quality meta-analyses that assessed the salivary flow rate of elderly people showed that the prevalence of hyposalivation in older people is higher than that in other populations, but there was no clear evidence to verify the relevance between the level of salivary production and function in elderly people (39, 40). Simultaneously, as a counterpart of the outcome, 1 systematic review of moderate quality showed that one of the reasons for oral changes in older people over 65 is long hospital stays or being institutionalized in a residential home (41), which therefore demonstrated the importance of developing protocols for elderly individuals' oral health care and providing more professional training to caregivers. Combined with the quality and results of the included reviews, malnutrition should be considered to be closely associated with poor oral health.

#### Malnutrition

Five reviews of low to high quality showed that the condition of the dentition is strongly associated with the prevalence of malnutrition in elderly individuals (28, 35–38), of which 2 quantitative meta-analyses of high quality pointed out that the nutritional status of elderly individuals with good dentition or dentures is significantly better than those with fewer functional tooth units or anodontia (28, 36). Meanwhile, 3 reviews of low to high quality demonstrated that masticatory problems, which have been identified as independent predictors for low BMI and serum albumin level, weight loss and protein-energy malnutrition (37), are also key for malnutrition (28, 35, 37). Elderly people with chewing problems had nearly twice the risk of malnutrition, and importantly, a lack of tooth/denture cleaning lack of autonomy for oral care, no access to the dentist and being edentulous placed elderly individuals at higher risk of malnutrition (28). On the other hand, two systematic reviews of moderate quality also showed that soft tissue problems, including tongue with blisters, dry or cracked lips and other functional loss, may hinder the intake of nutrients, which can also lead to malnutrition among older people (34, 37). Combined with the quality and results of the included reviews, malnutrition should be considered to be closely associated with poor oral health.

#### Frailty

The evidence for frailty in elderly individuals is complex. Two systematic reviews of moderate quality analyzed the factors including the number of teeth, chewing ability, oral function and the accumulation of oral health problems and showed that the prevalence of frailty, which is expected to increase rapidly in the aging population, is significantly related to oral health (29, 58), while 1 review of low quality that also included dental service use and Geriatric Oral Health Assessment Index scores claimed that poor oral health has not been evaluated longitudinally to show an association (57). This is because the assessment of frailty remains easily ignored in translational dental research and clinical practice (29). Therefore, combined with the quality and results of the included reviews, although evidence showed a positive association between oral health and frailty, the diversity of the included samples resulted in the outcome variable, and frailty should not be considered a clear outcome of poor oral health.

### Mental outcomes

Six systematic reviews provided data on mental outcomes (59–64), of which 1 included a meta-analysis (64). Five systematic reviews (none with a meta-analysis) focused on cognitive impairment (59–63), and 1 (with a meta-analysis) focused mainly on depression (64).

#### Cognitive impairment

One qualitative systematic review of low quality provided evidence of an association between learning and memory and complex attention and executive function and oral health in elderly individuals, of which most of the primary studies independently identified one or more significant unadjusted associations or adjusted covariates correlated with oral health (59). Correspondingly, the remaining 4 review articles of low to moderate quality focused on verifying the relation between oral health and dementia based on various oral assessment criteria (60–63). While one review of low quality showed that a few studies claimed that there was ambiguous evidence to demonstrate the association between oral health and dementia (63), most of the included studies reported that the risk of prevalence of orofacial pain, gingival bleeding or inflammation and dental caries is much higher in people with senile dementia (60–62). Together, these data suggest a positive effect of improving dental care and designing corresponding oral scales for earlier identification and effective interventions for preventing cognitive impairment in older people (59). Combined with the quality and results of the included reviews, cognitive impairment should be considered a possible outcome of poor oral health.

#### Depression

One systematic review with a meta-analysis of high quality studied the correlation between oral health and depression, which is the main cognitive disability in elderly individuals worldwide (64). The review quantitatively analyzed the outcome of oral diseases, where depression was considered the main potential result. Generally, although more longitudinal research to test causal and temporal relationships between depression and oral health status needs to be conducted, the existing results showed a positive association between depression and oral diseases in older people. Combined with the quality and results of the included reviews, depression should be considered a possible outcome of poor oral health.

### Social outcomes

Six systematic reviews provided data on social outcomes (44–49), of which 3 included a meta-analysis (46, 47, 49). The included reviews focused on analyzing the association between oral health and quality of life, which can be reflected by oral health-related quality of life (OHQoL) or the Geriatric Oral Health Assessment Index (GOHAI).

#### Quality of life

Four systematic reviews of moderate to high quality showed that a higher number of teeth, occluding pairs, implant-retained overdentures, and shortened dental arches were positively associated with OHQoL (45, 46, 48, 49), while xerostomia and orofacial pain were negatively related (44). Meanwhile, chewing is considered significantly relevant to talking, comfort, retention and aesthetics, which are crucial factors for improving quality of life (44, 48). Importantly, one review of moderate quality determined that there also exists a bidirectional relationship between oral health and diabetes mellitus, atherosclerosis, and cerebral vascular accidents (45). One meta-analysis of high quality reported a similar association in elderly individuals with Alzheimer's disease (49). Once the 2 meta-analyses of high quality quantitively analyzing the assessment of oral-related quality of life were included (46, 47), the data together showed that regular oral health care and utilizing dentures can greatly increase quality of life for older people. Combined with the quality and results of the included reviews, quality of life should be considered to be closely associated with poor oral health.

## Discussion

The aim of this umbrella review was to provide a systematic overview of the impacts of oral health on holistic health among elderly people, and summarize effective approaches for enhancing public oral health. According to the review and systematic synthesis of the included review papers (*N* = 35), the majority focused on multiple physical outcomes (*N* = 23); six papers mainly discussed mental outcomes, and 6 papers explored social outcomes. Correspondingly, clear evidence is available to support diverse outcomes covering physical, mental and social health in elderly individuals and included respiratory diseases, malnutrition, age-related oral changes, frailty, cognitive impairment, depression and poor quality of life. It is worth noting that although there were many commonalities among the reviews included, the perspectives of the papers varied, and none of the current articles provided a systematic summary of the diverse results, which is crucial for providing better health care for elderly individuals as well as reducing the cost of medical resources (28, 42, 50).

### Oral factors and global outcomes

Oral health is inseparable from holistic health and wellbeing, and the oral diseases are prevalent worldwide, which considerably placing a huge global burden and reducing the quality of life (65). Crucially, it is necessary to emphasize that this umbrella review showed that the oral health and holistic health of elderly individuals are strongly related. With aging, a decline in the physiology of elderly individuals is accompanied by the weakening of oral function and an increase in oral problems. Specifically, age-related oral changes can be summarized in three main aspects: (i) oral diseases such as dental caries and periodontitis resulting from bacterial colonization, which greatly increase the difficulty of oral cleaning (42, 43); (ii) tooth loss and chewing problems, which are closely associated with the diet of older people (29, 57, 58); and (iii) a decrease in salivary flow rate and oral soft issue function, which, however, have not been verified to affect the lives of the participants (39, 40, 61). Correspondingly, the changes were considered to physically lead to a range of impacts. As a consensus, microorganisms such as dental caries and periodontal pathogens colonized in saliva and dental plaque are the main potential risk factors for hospitalization and respiratory diseases, which significantly increase the incidence of aspiration pneumonia (13, 50, 55).

On the other hand, a complete lack of functional dentition and decreased masticatory ability can notably affect the selection and intake of food (35), e.g., fruits and vegetables containing various dietary fibers and vitamins, naturally resulting in malnutrition, including low BMI and serum albumin levels, weight loss and protein-energy malnutrition (28, 34–38). Additionally, the number of oral problems is positively related to the severity of malnutrition (37). In the meantime, poor oral health and oral-related physical diseases can directly or indirectly result in a multifaceted and complex condition of general health, i.e., frailty (29, 57, 58). Clearly, oral health is still considered to have a cumulative effect on frailty throughout life, which slowly contributes to the development of additional chronic conditions and leads to the deterioration of general health quickly in old age (57).

Similarly, in addition to physical outcomes, poor oral health can directly or indirectly affect the mental health of elderly individuals. Nangle's review clearly reported that oral health is significantly associated with cognitive conditions, including study, memory, complex attention, and executive function (59). Meanwhile, they also pointed out that there is still a lack of evidence to verify the relationship between poor oral health and the decrease in language and perceptual motor function in elderly individuals. Interestingly, the rest of the included reviews focusing on cognitive impairment mainly analyzed the impacts of oral health on older people with dementia (60–63). On the one hand, the prevalence of diseases of oral hard tissue (including dental caries, periodontitis and orofacial pain) and oral soft tissue (including gingival bleeding, periodontal pockets, stomatitis, mucosal lesions and reduced salivary flow) is significantly higher in elderly individuals with dementia than in people without dementia (60–62). One review also mentioned that some primary studies have found that oral health measures such as number of teeth and periodontal disease were associated with risk of cognitive decline or incident dementia, whereas others did not find an association (63); this needs further research. Importantly, one review of high quality showed a positive correlation between oral diseases and depression among elderly individuals over 65 years of age, probably because missing teeth hinder smiling, laughing or showing teeth without embarrassment (64). In addition, pain and difficulty speaking also leads to embarrassment and a loss of self-esteem contributing to loneliness and social isolation (66).

Finally, as a comprehensive and complex outcome, quality of life is also considered to be associated with oral health. Although there were different assessment criteria used in the included reviews, such as OHQoL, GOHAI, and OHIP (44–49), all the results supported the fact that some specific oral problems significantly impact quality of life. In particular, the number of teeth and chewing function were considered the main issues positively associated with quality of life (44, 45, 48). Moreover, oral problems can also lead to a host of socioeconomic problems; for example, receiving regular dental care or treatment of complications adds to the financial burden of older people, which is reflected by the generally lower OHQoL of socially deprived residents (45). However, a clearer map between diverse oral issues and quality of life requires more relevant research.

To summarize, the present results showed that oral health is significantly associated with holistic health in older people. Intuitively, poor oral condition can directly impact the physical health of elderly individuals and add to their mental burden or aggravate cognitive impairments; moreover, such physical and mental obstruction not only impacts quality of life socioeconomically but also worsens oral health. On the other hand, oral health has always been a neglected topic given low priority in contrast with many other competing health problems worldwide (67). As with most non-communicable diseases, oral conditions are chronic and strongly socially patterned, with elderly individuals being one of the groups affected (68). Therefore, to achieve significant improvements in oral health, effective strategies including oral health policy and guidelines are required to improve oral health of people with special health care needs. Good oral health can be established by early exposure to dental services, risk assessment and check-ups, as well as a meticulous oral health prevention regimen (7, 69).

### Solutions and suggestions

To establish a better relationship between oral health and holistic health, improvements can be summarized in the following three main aspects in public oral health.

(i) More rigorous and universal scales are needed. Although multiple outcomes of holistic health have been presented in a range of reviews and primary studies of high quality, it remains difficult to explain the relationship precisely. Moreover, popular scales are not always appropriate and may not fully represent actual oral health problems (49).

(ii) Providing oral hygiene and professional oral health care are important factors. Although evidence has demonstrated that routine oral care can achieve the same positive results (51, 53), professional methods are still considered effective measures to decrease the load of potentially pathogenic microorganisms and reduce the risk of aspiration pneumonia related to poor oral health (13, 15, 56). Especially for elderly individuals with limited mobility living in the community, nursing homes, and ICUs, professional oral health or routine oral hygiene care can not only reduce incidence of aspiration pneumonia and mortality rates due to oral-related diseases but can also result in significant cost savings in light of the economic burden of hospitalization to improve quality of life (50). Nurses should play a key role in referring elderly individuals to general practitioners or dental care providers (37). Attitudes and knowledge related to the screening, prevention and treatment of oral health problems need to be improved through the training and education of professional caregivers, which are important to eliminate the anxiety-related responses of dental fear among the elderly patients (70).

(iii) Improving self-awareness regarding oral care for elderly individuals is important. Oral problems are considered a cumulative process along with aging (57), and the older adults are generally at higher risk for dental infections and associated complex complications. Therefore, it is important for older people to clean their teeth regularly and use dentures once it becomes difficult to maintain their original dental habits (28, 29, 35, 36, 38, 57, 58). Simultaneously, the neatness and integrity of teeth is considered aesthetically attractive and is related to confidence in elderly individuals, which directly affects their mental and social wellbeing (64). Therefore, the importance of oral care should be emphasized more for elderly individuals during the nursing process.

In the process of screening the literature, we noticed some interesting primary studies verifying the relationship between oral health and other chronic diseases, such as diabetes (71), hypertension and metabolic syndrome; however, there was no corresponding systematic review, or the reviews did not focus on older people and therefore did not meet the screening criteria. In such cases, more cross-sectional and longitudinal research and corresponding systematic reviews should be conducted to help in the evaluation, prevention, intervention, and clinical practice of oral-related holistic health.

### Implications and limitations

There has been a rapid increase in research and corresponding systematic reviews on the impact of oral health on diverse chronic or non-chronic diseases in elderly individuals, which indicates the promotion of health awareness among elderly people in society. Accordingly, an umbrella review that qualitatively and quantitatively synthesizes studies by mixed methods provides an effective platform to efficiently extract relevant information for clinical practice and future research. Because the literature search was also systematic, according to the PRISMA guidelines, it gives a more comprehensive overview and a higher-level perspective than a narrative review with a narrow topic. Meanwhile, a convincing result of the umbrella review depends on the quality of the included systematic reviews with or without meta-analyses. Eleven of the 35 included reviews were of high quality.

In terms of the limitations, a more complete map of the final result in this research requires the inclusion of more studies written in languages other than English. Regarding the results of this study, although almost all of the included studies were associated with oral health, only one systematic review with 15 studies focused on depression and mental outcomes, which weakens the integrity of the conclusions. More importantly, it is worth noting that holistic health is a relatively broad concept, which indicates a wider result beyond the 7 exact outcomes in the present study. As a result, we are aware that the whole picture of the relationship between oral health and holistic health still has not been displayed through our analysis of the included papers, and that other symptoms or diseases remain to be further researched.

## Conclusion

This umbrella review provided a macro perspective to determine the multiple outcomes of holistic health associated with oral health and summarized intervention measures to improve public oral hygiene. On the basis of the consistent findings, clear evidence is available to show the multidimensional results of holistic health. Evidence indicates that oral health can be mainly divided into two issues: (i) poor oral hygiene, where the multiple pathogenic microorganisms colonizing the oral microbiome are significantly associated with respiratory disease, dental caries and periodontitis, and (ii) tooth loss and chewing problems, with which elderly individuals have a higher risk of malnutrition and frailty. The combination of the two issues can also result in poor mental health, such as cognitive impairment and depression, in older people. Correspondingly, to acquire a higher quality of life, elderly people are encouraged to clean their teeth regularly, to have dentures fitted and to receive timely oral care.

Among the included reviews, the majority provided a precise point of view to illustrate the results, while a few papers, especially those regarding mental and social outcomes, were more conservative in their discussions and argued that more research is needed, although evidence has shown significant correlations. In addition, it is necessary to emphasize that due to the lack of relevant systematic reviews, some other popular chronic oral-related diseases were not included in the present umbrella review. Although there are still some limitations and other different oral-related diseases remain to be studied, this umbrella review is expected to provide a global and effective reference for clinical practice in public health.

## Data availability statement

The original contributions presented in the study are included in the article/Supplementary material, further inquiries can be directed to the corresponding author/s.

## Author contributions

FL and XH led the current study. FL and SS initiated the study design and drafted the manuscript. FL, SS, and XH searched databases and screened the articles and extracted the data. XY and SH conducted the quality of the studies. XH, JH, and GW reviewed and revised the manuscript. All authors read and approved the final manuscript.

## Funding

This study was supported by Science & Technology Department of Sichuan Province (No. 2020JDKP0016).

## Conflict of interest

The authors declare that the research was conducted in the absence of any commercial or financial relationships that could be construed as a potential conflict of interest.

## Publisher's note

All claims expressed in this article are solely those of the authors and do not necessarily represent those of their affiliated organizations, or those of the publisher, the editors and the reviewers. Any product that may be evaluated in this article, or claim that may be made by its manufacturer, is not guaranteed or endorsed by the publisher.
