# Supplementary material for: Oral health-related multiple outcomes of holistic health in elderly individuals: An umbrella review of systematic reviews and meta-analyses
Source: Front Public Health. 2022 Oct 27;10:1021104. doi: 10.3389/fpubh.2022.1021104 (PMC9650948; doi:10.3389/fpubh.2022.1021104)
Supplement: Supplementary file 1 [file Table_1.DOCX]

Oral health-related multiple outcomes of holistic health in elderly individuals: An umbrella review of systematic reviews and meta-analyses

*Fan Liu^1^, Siping Song^2^, Xin Ye^3^, Shuqi Huang^2^, Jing He^,4^, Guan Wang^1^, and Xiuying Hu^5*^*

*^1^West China School of Nursing，Sichuan University / State Key Laboratory of Oral Diseases & National Clinical Research Center for Oral Diseases, West China Hospital of Stomatology**, Innovation Center of Nursing Research, Nursing Key Laboratory of Sichuan Province, West China Hospital, Sichuan University, Renmin South Road, Wuhou District, Chengdu, Sichuan Province, China*

*^2^Nursing department, State Key Laboratory of Oral Diseases & National Clinical Research Center for Oral Diseases, West China Hospital of Stomatology, Sichuan University，14 Renmin South Road, Wuhou District, Chengdu, Sichuan Province, China*

*^3^West China School of Nursing，Sichuan University, 17 Renmin South Road, Wuhou District, Chengdu, Sichuan Province, China*

*^4^Department of oral mucosal diseases, State Key Laboratory of Oral Diseases & National Clinical Research Center for Oral Diseases, West China Hospital of Stomatology, Sichuan University，14 Renmin South Road, Wuhou District, Chengdu, Sichuan Province, China*

*^5^Innovation Center of Nursing Research, Nursing Key Laboratory of Sichuan Province，West China Hospital, Sichuan University, 37 Guoxue Road, Wuhou District, Chengdu, Sichuan Province, China*

*** Correspondence:**

Xiuying Hu

[huxiuying@scu.edu.cn](mailto:huxiuying@scu.edu.cn)

## Supplementary Methods

## Checklist of JBI Critical Appraisal Checklist for Systematic Reviews and Research Syntheses

Q1. Is the review question clearly and explicitly stated?

Q2.Were the inclusion criteria appropriate for the review question?

Q3.Was the search strategy appropriate?

Q4. Were the sources and resources used to search for the studies adequate?

Q5. Were the criteria for appraising studies appropriate?

Q6. Was the critical appraisal conducted by two or more reviewers independently?

Q7. Were there methods to minimize errors in data extraction?

Q8. Were the methods used to combine studies appropriate?

Q9. Was the likelihood of publication bias assessed?

Q10.Were recommendations for policy and practice supported by the reported data?

Q11.Were the specific directives for new research appropriate?

*If the evaluation is low risk, the score is 1, and high risk or unclear is 0.*

## Supplementary Tables

### Supplementary Table S1. Keywords and search strategy in the umbrella review.

| **Database** | **Keywords** |
| --- | --- |
| PubMed | 1. Dental Care  2. Dental Care for Aged  3. 1 or 2  4. Oral Health  5. Oral Hygiene  6. 4 or 5  7. 3 and 6  8. Meta-Analysis as Topic/ or meta-analys*.mp.  9. 7 and 8 |
| MEDLINE | 1. Dental Care  2. Dental Care for Aged  3. 1 or 2  4. Oral Health  5. Oral Hygiene  6. 4 or 5  7. 3 and 6  8. Meta-Analysis as Topic/ or meta-analys*.mp.  9. 7 and 8 |
| Web of Science | 1. Dental Care  2. Dental Care for Aged  3. 1 or 2  4. Oral Health  5. Oral Hygiene  6. 4 or 5  7. 3 and 6  8. Meta-Analysis as Topic/ or meta-analys*.mp.  9. 7 and 8 |
| Cochrane Library | 1. Dental Care |
|  | 2. Dental Care for Aged |
|  | 3. 1 or 2 |
|  | 4. Oral Health |
|  | 5. Oral Hygiene |
|  | 6. 4 or 5  7. 3 and 6 |
|  | 8.Meta-Analysis as Topic/ or meta-analys*.mp. |
|  | 9. 7 and 8 |

### Supplementary Table S2. Quality appraisal of the included reviews

| **study** | **Q1** | **Q2** | **Q3** | **Q4** | **Q5** | **Q6** | **Q7** | **Q8** | **Q9** | **Q10** | **Q11** | **Sum** | **QoE** |
| --- | --- | --- | --- | --- | --- | --- | --- | --- | --- | --- | --- | --- | --- |
| Algra 2021 | 1 | 1 | 1 | 1 | 1 | 1 | 1 | 1 | 0 | 0 | 1 | 9 | 3 |
| Hussein/2021 | 1 | 1 | 1 | 1 | 1 | 1 | 1 | 1 | 1 | 1 | 1 | 11 | 4 |
| Tada/2014 | 1 | 1 | 1 | 1 | 1 | 1 | 0 | 1 | 0 | 0 | 1 | 8 | 2 |
| Toniazzo/2017 | 1 | 1 | 1 | 1 | 1 | 1 | 1 | 1 | 1 | 0 | 1 | 10 | 4 |
| Lancker/2012 | 1 | 1 | 1 | 1 | 1 | 1 | 1 | 1 | 0 | 0 | 1 | 9 | 3 |
| Zelig/2000 | 1 | 1 | 1 | 1 | 1 | 1 | 1 | 1 | 1 | 1 | 1 | 11 | 4 |
| Affoo/2015 | 1 | 1 | 1 | 1 | 0 | 1 | 1 | 1 | 0 | 0 | 1 | 8 | 4 |
| Pina/2020 | 1 | 1 | 1 | 1 | 1 | 1 | 1 | 1 | 1 | 1 | 1 | 11 | 4 |
| Ruiz-roca2021 | 1 | 1 | 1 | 1 | 1 | 1 | 1 | 1 | 1 | 1 | 1 | 11 | 3 |
| Chan2021 | 1 | 1 | 1 | 1 | 1 | 1 | 1 | 1 | 0 | 0 | 1 | 9 | 3 |
| López/2017 | 1 | 1 | 1 | 1 | 0 | 0 | 0 | 1 | 0 | 0 | 1 | 6 | 2 |
| van de Rijt, MSc/2019 | 1 | 1 | 1 | 1 | 1 | 1 | 1 | 1 | 0 | 0 | 1 | 9 | 3 |
| Wong/2019 | 1 | 1 | 1 | 1 | 1 | 1 | 1 | 1 | 0 | 0 | 1 | 9 | 3 |
| Azami-Aghdash /2021 | 1 | 1 | 1 | 1 | 0 | 1 | 1 | 1 | 0 | 0 | 1 | 8 | 4 |
| Baniasadi/2021 | 1 | 1 | 1 | 1 | 1 | 1 | 1 | 1 | 1 | 0 | 1 | 10 | 4 |
| De Medeiros2019 | 1 | 1 | 1 | 1 | 1 | 1 | 1 | 1 | 0 | 0 | 1 | 9 | 3 |
| Ming2019 | 1 | 1 | 1 | 1 | 1 | 1 | 1 | 1 | 0 | 0 | 1 | 9 | 4 |
| Azarpazhooh/2006 | 1 | 1 | 1 | 1 | 1 | 1 | 1 | 1 | 0 | 1 | 1 | 10 | 3 |
| Khadka/2021 | 1 | 1 | 1 | 1 | 1 | 1 | 1 | 1 | 0 | 0 | 1 | 9 | 3 |
| Liu C 2018 | 1 | 1 | 1 | 1 | 1 | 1 | 1 | 1 | 1 | 1 | 1 | 11 | 4 |
| Loeb/2003 | 1 | 1 | 1 | 1 | 1 | 1 | 1 | 1 | 1 | 0 | 1 | 10 | 3 |
| Scannapieco/2003 | 1 | 1 | 1 | 1 | 1 | 1 | 1 | 1 | 0 | 1 | 1 | 10 | 3 |
| Sjögren/2016 | 1 | 1 | 1 | 1 | 1 | 1 | 1 | 1 | 0 | 1 | 1 | 10 | 4 |
| Sjögren/2008 | 1 | 1 | 1 | 1 | 1 | 1 | 1 | 1 | 0 | 1 | 1 | 10 | 3 |
| van der Maarel-Wierink C/2011 | 1 | 1 | 1 | 1 | 1 | 1 | 1 | 1 | 1 | 1 | 1 | 11 | 3 |
| van der Maarel-Wierink / 2013 | 1 | 1 | 1 | 1 | 1 | 1 | 0 | 1 | 0 | 0 | 1 | 8 | 2 |
| Slashcheva2021 | 1 | 1 | 1 | 1 | 1 | 1 | 0 | 1 | 0 | 1 | 1 | 9 | 3 |
| Tôrres2015 | 1 | 1 | 1 | 1 | 1 | 1 | 0 | 1 | 0 | 0 | 1 | 8 | 2 |
| Hakeem/2019 | 1 | 1 | 1 | 1 | 1 | 1 | 1 | 1 | 0 | 1 | 1 | 10 | 3 |
| Nangle2019 | 1 | 1 | 1 | 1 | 0 | 0 | 1 | 1 | 0 | 0 | 1 | 7 | 2 |
| Lauritano/2019 | 1 | 1 | 1 | 1 | 1 | 1 | 1 | 1 | 0 | 0 | 1 | 9 | 3 |
| Delwel /2018 | 1 | 1 | 1 | 1 | 1 | 1 | 1 | 1 | 0 | 1 | 1 | 10 | 3 |
| Delwel2017 | 1 | 1 | 1 | 1 | 1 | 1 | 1 | 1 | 0 | 1 | 1 | 10 | 3 |
| Wu/2016 | 1 | 1 | 1 | 1 | 0 | 0 | 1 | 1 | 0 | 0 | 1 | 7 | 2 |
| Cademartori/2018 | 1 | 1 | 1 | 1 | 1 | 1 | 1 | 1 | 0 | 0 | 1 | 9 | 4 |

Abbreviations: QoE = quality of evidence

### Supplementary Table S3. Summary of evidence for oral health related multiple outcomes of holistic health and statistically significant findings shown in bold.

| **study** | **oral evaluation** | **diagnostic criteria / symptom descriptions of outcomes measurement / definition of outcomes** | **Positive-oral-related- findings** | **Negative-oral-related findings** | **JBI** | **QoE** |
| --- | --- | --- | --- | --- | --- | --- |
| **NUTRITION** | | | | | | |
| Algra 2021  Systematic review | Objective oral health: ROAG, ROAG-J, OHAT, DMFT/DFT, FTUs, stimulated salivary flow;  Subjective oral health: GOHAI, self-administered or standardized questionnaires, OHIP, xerostomia, chewing problems and oral hygiene. | SGA, MNA, MNA-SF, BMI, weight loss, MNA+BMI / weight loss, BMI+ unintentional weight loss / time-specific weight loss, sarcopenia. | 2 studies showed the malnourished elderly had **significantly less** FUs (＜4) or FTUs (8.3 ±1.1).  4 studies reported **higher proportion** of soft tissue problems, including tongue with blisters, and dry or cracked lips in malnourished participants.  7 studies showed malnutrition was associated with subjective oral health, such as chewing pain, autonomy of oral care, and negative self-perception of oral health **(p＜0.05)**.  4 studies demonstrated the association between (stimulated) **low flow** or xerostomia and malnutrition. | 1 study showed **no significant** difference in FTUs in sarcopenia.  2 studies showed there was **no significance** between nutritional status both DMFT and prosthetic status (p＞0.05).  1 study demonstrated **non-significant** associations between malnutrition and xerostomia. | 9 | 3 |
| Hussein/2021 | Dental status: number of teeth present/lost, edentulism, use of dental prosthesis, occlusion;  oral health indexes: GOHAI, OHIP-14, DFT, ROAG, dental plaque;  oral health conditions: difficulty chewing, dry mouth. | MNA (0-30 points): > 24 indicates well-nourished, 17-23.5 suggests at-risk for malnutrition and <17 indicated malnourishment  MNA-SF (0-15 points): 12-14 indicates no risk, 8-11 indicates at-risk for malnutrition, | The lack of daily oral hygiene (teeth or denture cleaning), chewing problems and being partially/fully edentulous, **put older adults at higher risk** of malnutrition;  8 studies showed partial or fully edentulous elderly had a **9.5% higher** risk of malnutrition (RR=1.095; 95%CI 1.007-1.190; p=0.033);  9 studies demonstrated older adults with a prosthesis had 3.7% lower risk of malnourishment (RR=0.963; 95%CI 0.862-1.076; p=0.505);  3 studies figured older adults with chewing problems had nearly **twice** risk of malnutrition (RR=1.956; 95%CI 1.097-3.488; p=0.023).  2 studies showed older people with no daily teeth or denture cleaning were at **52.6% higher** risk of malnutrition (RR=1.526; 95%CI 1.261-1.847; p＜0.001);  Poor/moderate oral health (GOHAI score<57), lack of autonomy for oral care, no access to the dentist and being edentulous were at **high risk** of malnutrition (p<0.001). | 2 studies showed At-risk or malnourished individuals had on average lost 4 more teeth was **not statistically** significant (mean difference =-3.858; 95%CI -7.968=-0.252; p=0.066);  3 studies showed **no statistically** significant difference based on MNA/MNA-SF for oral health. | **11** | **4** |
| Tada/2014 | masticatory ability, and MAFs, including number of teeth, number of occlusion pairs, dentition status (dentate or edentate), dentition adequacy (adequate or inadequate), and denture status. | Self-reported or interviewed food intake; nutrient intake calculated from food intake; nutrient profile measured on serum and blood. | In 22/28 cross-sectional studies, older people with better mastication and dentition reported **significantly higher** consumption of foods and intake of nutrients than those with poorer oral health;  6 studies demonstrated a **significant** correlation between masticatory ability and food intake, foods with significance in intake between high and low masticatory ability included fruit and vegetables. The major nutrients with **significant** differences in intake included dietary fiber and vitamins. | In 6/28 studies showed **no significance** between mastication and dentition.  Five (5/7) intervention studies did **not show** significant improvement in food and/or nutrient intake with new prostheses. | 8 | 2 |
| Toniazzo/2017 | Edentulous, use of prosthesis, mean number of present teeth, functional teeth units or occluding pairs, DMFT, dental plaque. | Nourished (Mini Nutritional Assessment, MNA<17), at risk of malnutrition (MNA 17-24), MNA-SF | Well-nourished subjects had a **significantly higher** number of pairs of FTUs.  5 studies showed that FTU and mean number of present teeth were **significantly associated** with nutritional status (SMD: -0.141; 95%CI -0.278 - -0.005, p=0.042) | **No statistically** significant association between edentulism (RR=1.072; 95%CI 0.957-1.200, p=0.230) and use of prosthesis (RR=0.874; 95%CI 0.710-1.075, p=0.202) in 8 and 4 studies, respectively. | **10** | **4** |
| Lancker/2012 | Number of natural teeth, edentulism, denture use, chewing ability, ROAG, oral candidiasis. | BMI, serum albumin level, weight loss, MNA, SGA, NuSc | 4 studies showed masticatory problems were identified as the **independent predictor** for low BMI and level of serum albumin, weight loss and protein energy malnutrition by multivariate analysis (OR: 1.01-1.52);  1 study reported dental condition was **association with** malnourished (OR 3.00; 95%CI 1.12-8.06, p<0.05);  2 studies frond a **significantly** association between the number of oral problems and malnutrition. | Caution is needed for the interpretation of these results because of the absence of a gold standard to define and assess malnutrition and oral health status and the presence of methodological limitations throughout the studies. | 9 | 3 |
| Zelig/2020 | tooth loss (<28 teeth) or tooth replacement (removable full or partial dentures, implants dentures). | Using 1 of the 4 approved validated nutrition screening or assessment tools: MUST, MST, MNA, and the SGA. | 6 studies revealed older adults who were completely or lack of functional dentition had a **21% increased** risk of malnutrition (RR=1.21; 95%CI 1.11-1.32). | NA | **11** | **4** |
| **AGE-RELATED ORAL CHANGES** | | | | | | |
| Affoo/2015 | Aging process | Whole salivary flow (whole/unstimulated whole), SMSL salivary flow (all/unstimulated SMSL), parotid salivary flow, minor gland salivary flow. | Whole salivary flow rate and SMSL salivary flow rate were **significantly** lower in elderly group, SMD=0.551, SE=0.056; SMD=0.611, SE=0.075, respectively. | 21 studies reported no significant differences in mean salivary flow between younger and older subjects. | 8 | 4 |
| Pina/2020 | Aging process | Overall hyposalivation prevalence. | Overall hyposalivation prevalence of 33.37% (95% CI: 23.90-43.57, P <0.01), prevalence of hyposalivation for unstimulated and stimulated methods was 33.39% (95% CI 21.08-46.96, P <0.001) and 30.47% (95% CI 22.53-39.04, p< 0.001), respectively. | Salivary flow reduction is **not necessarily** associated with the subjective feeling of dry mouth and other xerostomia problems. | 11 | 4 |
| Ruiz-roca2021 | DMFT, OHAT, bacterial plaque and mucosal status, halitosis | DMFT, OHAT, dentist and nurse to measure halitosis | The oral health of patients (≥65 y) is **worse than** that of the rest population. Long hospital stays or being institutionalized in a residence makes this group susceptible to a worsening of their oral health status. | NA | 11 | 3 |
| Chan2021 | Aging process | DMFT and DFR | The prevalence of root caries ranged from 8% (Finland) to 74% (Brazil), while from 25% (Australia) to 99% (South Africa) in community dwellers. The situation was even **worse in** institutionalised older adults of which the mean DMFT score varied from 6.9 (Malawi) to 29.7 (South Africa) | NA | 9 | 3 |
| López/2017 | Changes in age | Main risk factors for periodontitis and their likely influence on the future periodontitis burden in the elderly are discussed. | Risk indicators for root caries include caries experience, the number of surfaces at risk and poor oral hygiene. | NA | 6 | 2 |
| **QUALITY OF LIFE** | | | | | | |
| van de Rijt, MSc/2019 | Dentate, edentate, number of missing /teeth/occluding pairs, DT, DMFT, FT, retained roots, pocket depth, mobility, bleeding, gingivitis, AOM, IOD, conventional denture, partial denture, denture treatment need, xerostomia, chewing function. | Quality of life is defined as “an individual’s perception of their position in life in the context of the culture and value systems in which they live and in relation to their goals, expectations, standards and concerns”. (WHO) | OHQoL in people aged 65 years or older is **positively associated** with higher number of teeth, higher number of occluding pairs, implant-retained overdentures, and the shortened dental arch concept and negatively associated with xerostomia, orofacial pain, and poor chewing ability. | There is **no consensus** on the association between edentulism, caries, and periodontal conditions, use of dentures, hyposalivation and OHQoL. | 9 | 3 |
| Wong/2019 | DMFT Index, RCI, decayed root, ROAG, visual plaque index, community periodontal index. | OHRQoL | Periodontitis, decayed, missing or filled teeth >20, mucosal lesions **were associated** with a poor OHRQoL, socially deprived residents or those with mild or above cognitive impairment.  Those with a poor OHRQoL might **show signs** of poor nutrition. | The relationship between oral health, the OHRQoL and nutrition in this at-risk population also warrants exploration. | 9 | 3 |
| Azami-Aghdash /2021 | Oral health | GOHAI: 0-60 points, OHIP-14: 0-70 points, higher scores are indicating the quality of life associated with oral and dental health.  OHIP-49: 0-100 points, 0 score indicates the high level of life quality associated with oral and dental hygiene. | Total average of life quality in the elderly group is 80.2 for GOHAI; The total average of life quality associated with oral and dental hygiene in the elderly group is 14.8; | The elderly group of the population had no proper oral health-related quality of life. | 8 | 4 |
| Baniasadi/2021 | Generic QoL screening tool, OHIP, caries history, tooth-induced pain, DMFT scores and DMFT scores/DMFS scores | GOHAI | A **positive association** between low educational level, marital status, depression, smoking status, denture wearing, poor general health, tooth-induced pain, periodontal diseases and poor OHRQoL among the elderly. | **A negative** association between DMFT, being older than 75 years of age on poor OHRQoL among the elderly. | 10 | 4 |
| De Medeiros2019 | OHIP, OIDP | GOHAI | Complete denture treatment **improves** elderly patients’ QoL after denture replacement, satisfaction and QoL in treated patients were related to the ability to chew and talk, comfort, and esthetics. | The limited methodologic quality in the reported studies underscores the need for more robust controlled investigations to strengthen the current body of evidence. | 9 | 3 |
| Ming2019 | OHIP | GOHAI, OHRQoL | 3 studies reported **higher** **OHRQoL scores** with AD than those among controls.  3 studies reported that prosthetic type and quality **positively affected** OHRQoL among participants with AD. | OHRQoL may **not fully** represent actual oral health problems of patients with AD.  4 studies were found **no significant** differences in GOHAI scores between participants with AD and controls. | 9 | 4 |
| **RESPIRATORY DISEASES** | | | | | | |
| Azarpazhooh/2006 | Oral health indicators and oral intervention. | Examined the efficacy of improvement of oral health indicators in reducing the incidence and occurrence of pneumonia. | 3 studies found the presence of cariogenic and periodontal pathogens in saliva and dental plaque **(OR = 4 to 9.6)** and dental decay **(OR=1.2)** were the potential risk factors for pneumonia.  1 study showed **higher plaque scores** were associated with a previous history of respiratory tract infection.  4 studies showed a **potential association** between periodontal disease and COPD. | NA | 10 | 3 |
| Khadka/2021 | Determination of oral health focused on number of teeth present, number with dental caries, depth of periodontal pockets, moistness of oral mucosa and tongue, chewing and swallowing difficulty. | Diagnosis of aspiration pneumonia varied and was based on fever ≥37.8℃, number of febrile days, chest X-radiographs (findings areas of increased density), auscultatory findings, cough, sputum, dyspnea or pleuritic chest pain or positive pleuritic fluid cultures and white blood cell count >5,000 cells/mm3. | 12 studies reported colonization of the oral cavity of older people by microorganisms commonly **associated with** aspiration pneumonia.  Aspiration pneumonia **occurred less** in people who received professional oral care.  Isolation of Candida albicans, Staphylococcus aureus, methicillin-resistant S. aureus and Pseudomonas aeruginosa was **related to mortality** due to aspiration pneumonia. | NA | 9 | 3 |
| Liu C 2018 | Oral interventions | Mortality (pneumonia-associated, 24 moths of followed up), mortality (all-cause), prevalence of fever. | 2 studies showed that professional oral care may **reduce** the risk of pneumonia-associated mortality. | 1 study was unable to determine whether professional oral care resulted in lower incidence rate of NHAP compared with usual oral care over an 18-month period (hazard ratio 0.65, 95% CI 0.29 to 1.46).  1 study was **unable to determine** whether professional oral care resulted in a lower number of first episodes of pneumonia compared with usual care over a 24-month period (RR 0.61, 95% CI 0.37 to 1.01). | 11 | 4 |
| Loeb/2003 | Oral interventions | The prevention of aspiration pneumonia | 1 RCT conducted in residents of nursing homes: oral care--nurses or caregivers cleaned the teeth of residents after every meal with an applicator of povidone iodine, no active treatment. pneumonia was noted in 21 of 184 (11%) patients assigned to oral hygiene versus 34 of 184 (19%) patients who received no oral care (OR=1.74, 95%cl 0.93-3.26) | Insufficient data exist to determine the effectiveness of positioning strategies, modified diets, oral hygiene, feeding tube placement, or delivery of food in preventing AP. | 10 | 3 |
| Scannapieco/2003 | Clinical periodontal status, panoramic radiological status, number of carious teeth, dental plaque, calculus, oral hygiene status, and buccal mucosa. | For cohort studies that measured differences in rates of disease between groups with and without oral disease, weighted mean differences, relative risks, or odds ratios were compared. A meta-analysis was performed on the 5 intervention studies to determine the relationship between oral hygiene intervention and rate of pneumonia in institutionalized patients. | Oral interventions improving oral hygiene through mechanical and/or topical chemical disinfection or antibiotics **reduced the incidence** of nosocomial pneumonia by an average of 40%.  Oral colonization by respiratory pathogens, fostered by poor oral hygiene and periodontal diseases, appears to be **associated with** nosocomial pneumonia. | 5 oral hygiene intervention trials showed that oral hygiene intervention **significantly reduced** the odds of pneumonia and dramatically **reduce the rate** of pneumonia in institutionalized subjects (OR 3.00, 95%CI 2.06-4.37). | 10 | 3 |
| Sjögren/2016 | Oral care interventions given by 1) dental personnel (dental hygienist or dentists), 2) by nursing personnel 3) by dental or nursing personnel | Mortality from healthcare-associated pneumonia in elderly adults in hospitals or nursing homes | 2 studies showed oral care interventions given by dental personnel **reduced** mortality from HAP (RR=0.43, 95%CI 0.25-0.76). | 3 studies showed oral care interventions given by nursing personnel **was not** statistically significant in mortality from HAP (RR = 1.2, 95% CI 0.97 - 1.48).  5 RCTs showed intensified oral care interventions given by dental or nursing personnel was slightly protective against mortality from HAP (RR = 0.80, 95% CI = 0.49–1.31). | 10 | 4 |
| Sjögren/2008 | Oral care interventions | The preventive effect of oral hygiene on pneumonia and respiratory tract infection. | 4 RCTs revealed **positive preventive** effects of oral hygiene on pneumonia and respiratory tract infection in hospitalized elderly people and elderly nursing home residents with absolute risk reductions from 6.6% to 11.7% and needed to treat from 8.6 to 15.3 individuals. | 10 non-RCT studies contributed to **inconclusive evidence** on the association and correlation between oral hygiene and pneumonia or respiratory tract infection in elderly people. | 10 | 3 |
| van der Maarel-Wierink C/2011 | An oral assessment guide score, assessing swallow, lips, tongue, saliva, mucous membranes, gingiva, and teeth or dentures. | The risk of aspiration pneumonia | Several bad oral health factors seem to **play a role** in the risk of aspiration pneumonia in frail older people.  In 4 cohort studies, various bad oral health factors showed to be risk factors for aspiration pneumonia. | NA | 11 | 3 |
| van der Maarel-Wierink / 2013 | Oral health care | The effect on the incidence of aspiration pneumonia. | 2 studies showed that improvement of oral health care **diminished** the risk of developing aspiration pneumonia and the risk of dying from aspiration pneumonia directly.  3 studies showed that adequate oral health care **decreased** the amount of potential respiratory pathogens and suggested a **reduction** in the risk of aspiration pneumonia by improving swallowing reflex and cough reflex sensitivity. | NA | 8 | 2 |
| **FRAILTY** | | | | | | |
| Slashcheva2021 | Dysphagia, oral examination, self-reported oral health questionnaire (OHIP, EuroQol-5D), number of teeth, chewing pain, maximal occlusal force, mixing ability, chewing ability, GOHAI. | Oral frailty questionnaire, self-reported frailty checklist, OSPHE frailty phenotype, fried frailty criteria, Frailty Index. | Frailty prevalence ranged from **8.5% to 66.0%**.  25 studies demonstrated **significant** covariate-adjusted association between frailty status and number of teeth, chewing ability, prosthetic characteristics, dental caries, periodontitis, dental utilization and oral health-related quality of life factors. | Despite robust evidence of association with oral health characteristics, frailty assessment has yet to **be sufficiently** applied to translational dental research and clinical practice. | 9 | 3 |
| Tôrres2015 | Number of teeth, masticatory ability, occluding pairs of teeth, dental prosthesis, periodontal disease, dental service use, and GOHAI | Frailty is defined as a combination of biological, physiological, social, and environmental changes that occur with advancing age.  Fried Frailty Index, Fatigue Scale, Weight loss, Handgrip strength | 2 studies that used the FFI reported the prevalence of frailty ranged from 8.5% (n = 117) for a Brazilian population to 15% (n = 105) for a Mexican population.  Predictors and covariates found in the studies were number of teeth, masticatory ability, dental prosthesis, dental service use, self-report of oral health, and GOHAI. | **None of** the studies that were evaluated longitudinally showed whether poor oral health increases the likelihood of developing signs of frailty, although the studies suggest that there may be an association between frailty and oral health. | 8 | 2 |
| Hakeem/2019 | Oral health indicators were number of teeth, periodontal disease, oral functions (functional dentition with occluding pairs and maximum bite force), use of removable dentures, accumulation of oral health problems and dry mouth symptoms. | Frailty indicated by any validated scale or measurement or index (eg, Frailty Index, Fried's frailty criteria based on phenotype model or Edmonton Frailty Scale). | The studies showed **significant association** of number of teeth (2 studies), oral functions (2 studies), accumulation of oral health problems and number of dry mouth symptoms with frailty incidence. | whereas **periodontal disease showed** inconsistent associations with frailty incidence. | 10 | 3 |
| **COGNITIVE IMPAIRMENT** | | | | | | |
| Nangle2019 | periodontal status –CPITN; *Porphyromonas gingivalis* IgG; | DSST; Serial subtraction; Memory subscales from MMSE; Immediate and delayed verbal and visual recall and recognition; prospective memory; Visual and verbal paired associates; recognition; Block design; Constructional praxis; National Adult Reading Test; Verbal fluency | The study provides evidence of **an association** between learning and memory, complex attention, and executive function with oral health in old age. Gaining a detailed picture of how specific types of cognitive decline relate to oral health has potential implications for earlier identification of older adults who experience oral health problems, and inform the development of more effective interventions focused on enhancing oral health outcomes. | Results were less clear for the domains of language and perceptual motor function. No study assessed the relationship between social cognition and oral health. | 7 | 2 |
| Lauritano/2019 | Oral Health Index; DMFT; Periodontal Index, Salivary flow | DSM-III and IV; ICD-9 and 10; NINCDS-ADRDA; MMSE; computer tomography; MRI; CDR; Abbreviated Mental Test. | Coronal/root caries and retained roots were **more common** in people with dementia than in those without dementia. Most of the participants with dementia presented gingival bleeding or inflammation and they suffered from the periodontal disease more than people without dementia. | **No significant** differences were found between both groups with regard to the number of present teeth, DMFT Index, edentulousness/use of denture, and orofacial pain. | 9 | 3 |
| Delwel /2018 | Bleeding Index, Community Periodontal Index, clinical attachment level, Debris Index, Drug-induced xerostomia, daily oral health care, Gingival Bleeding Index, Plaque Index, Presence of deposits, Presence of calculus/plaque/gingival bleeding, Presence of gingivitis and periodontal disease, probing pocket depth, presence of visible dental plaque, Presence of debris, presence of inflamed, mucosal pathology, mouth dryness, salivary flow, Simplified Oral Hygiene Index of Greene and Vermillion | The diagnosis of dementia was considered adequate if the following criteria for dementia diagnosis were used: DSM-III and IV, ICD-9 and 10, and the Alzheimer’s criteria by the National Institute of Neurological and Communicative Disorders and Stroke and NINCDS-ADRDA | The elderly with dementia has **high levels** of plaque and **many oral** health problems related to oral soft tissues, such as gingival bleeding, periodontal pockets, stomatitis, mucosal lesions, and reduced salivary flow. | NA | 10 | 3 |
| Delwel2017 | GOHAI | DSM-III or IV; ICD-10; NINCDS-ADRDA; CT; MRI; PET; CDR; MHLW; AMT; MMSE; MDS-COGS. | The elderly with dementia has **worse** oral health, with more retained roots and coronal and root caries, when compared to older people without dementia. | **An equivalent prevalence** of orofacial pain, number of teeth present, decayed missing filled teeth index, edentulousness percentage, and denture use was found for both groups. | 10 | 3 |
| Wu/2016 | The number of teeth, periodontal and caries problems, and denture use. | Cognition was most frequently evaluated using the Mini-Mental State Examination or according to a diagnosis of dementia. | Some studies found that oral health measures such as number of teeth and periodontal disease were associated with risk of cognitive decline or incident dementia, whereas others did not find an association. Similarly, cognitive decline was not consistently associated with greater loss of teeth or number of decayed teeth. | It is **unclear** how or whether oral health and cognitive status are related. | 7 | 2 |
| **DEPRESSION** | | | | | | |
| Cademartori/2018 | Any tooth loss or edentulism, periodontal disease, and dental caries. | As criteria for a diagnosis of depression, studies with a primary diagnosis of depression, including clinical diagnoses or diagnostic criteria through validate tools, were selected. Other mental disorders and dental phobias were not included, GDS, CIDI, PHQ-8, CES-D, self-report. | When the oral diseases were tested as **independent variable** and depression as outcome (4 studies), associations with both edentulism (OR 1.28; 95% CI 1.06-1.55) and periodontal disease (HR 1.73; 95% CI 1.58-1.89) were found, showing a **positive** association between depression and oral diseases, specifically dental caries, tooth loss, and edentulism, in adults and elders. | More longitudinal studies are required to test causal and temporal relationship between depression and oral health status. | 9 | 4 |

Abbreviations: AD = Alzheimer's disease; AMT = abbreviated mental test; AOM = abnormalities oral mucosa; BDI = Beck’s depression inventory; BMI = body mass index; CES-D = center for epidemiological studies-depression scale; CIDI = composite international diagnostic interview; CDR = clinical dementia rating; COPD = chronic obstructive pulmonary disease; CPITN = community periodontal index of treatment needs; DFT = decayed/filled teeth; DFR = decayed and filled root; DMFS = decayed/missing/filled permanent surfaces; DMFT = decayed/missing/filled teeth; DSM = diagnostic and statistical manual of mental disorders; DSST = digit symbol substitution test; DT = decayed teeth; FUs = (posterior dental) functional units; FTUs = functional teeth units;

GDS = geriatric depression scale; GOHAI = geriatric oral health assessment instrument;

HAP = healthcare-associated pneumonia; ICD = international classification of disease;

IOD = implant-retained overdenture; MAFs = mastication associated factors; MCI = mild cognitive impairment; MDS-COGS = minimum data set cognitive score; MHLW = ministry of health, labor, and welfare; MMSE = mini mental state examination; MNA = mini nutritional assessment; MNA-SF = MNA short form; MRI = magnetic resonance imaging; MST = malnutrition screening tool; MUST = malnutrition universal screening tool; NA = not available; NHAP = nursing home-acquired pneumonia; NINCDS-ADRDA = national institute of neurological disorders and stroke Alzheimer’s disease and related disorders association; NuSc = nutritional score; OHAT = oral health assessment tool; OHIP = oral health impact profile; OHQoL = oral health-related quality of life; OIDP = oral impact on daily performance; PET = positron emission tomography; PHQ-8 = patient health questionnaire 8; PI-LTC = combined plaque index for long-term care; PSR = periodontal screening and recording; RCI = root caries index; RCT = randomized controlled trials; ROAG = revised oral assessment guide; ROAG-J = revised oral assessment guide -Jönköping; RR = relative risk; SE = standard errors; SGA = subjective global assessment; SMD = standard mean difference; SMSL = submandibular and sublingual; WHO = World Health Organization
